# The cinema-cognition dialogue: a match made in brain

**DOI:** 10.3389/fnhum.2012.00248

**Published:** 2012-09-04

**Authors:** Yadin Dudai

**Affiliations:** Department of Neurobiology, The Weizmann Institute of ScienceRehovot, Israel

**Keywords:** brain, cinema, dissociative states, emotional mental travel, mental time travel, working memory

## Abstract

That human evolution amalgamates biological and cultural change is taken as a given, and that the interaction of brain, body, and culture is more reciprocal then initially thought becomes apparent as the science of evolution evolves (Jablonka and Lamb, 2005). The contribution of science and technology to this evolutionary process is probably the first to come to mind. The biology of *Homo sapiens* permits and promotes the development of technologies and artefacts that enable us to sense and reach physical niches previously inaccessible. This extends our biological capabilities, but is also expected to create selective pressures on these capabilities. The jury is yet out on the pace at which critical biological changes take place in evolution. There is no question, however, that the kinetics of technological and cultural change is much faster, rendering the latter particularly important in the biography of the individual and the species alike. The capacity of art to enrich human capabilities is recurrently discussed by philosophers and critics (e.g., Arsitotle/*Poetics*, Richards, 1925; Smith and Parks, 1951; Gibbs, 1994). Yet less attention is commonly allotted to the role of the arts in the aforementioned ongoing evolutional tango. My position is that the art of cinema is particularly suited to explore the intriguing dialogue between art and the brain. Further, in the following set of brief notes, intended mainly to trigger further thinking on the subject, I posit that cinema provides an unparalleled and highly rewarding experimentation space for the mind of the individual consumer of that art. In parallel, it also provides a useful and promising device for investigating brain and cognition.

## On the cinema-brain resonance

Born just a little over a century ago, cinema capitalized on the rich history of the art of the theatre and on developments in the technology of photography, while harnessing the visual illusion of motion. Combined with the budding of globalization, this culminated in the fast development of cinema into a popular cognitive domain and social phenomenon, and ultimately into a rich universe of visual (and ultimately audiovisual) artistic and social experience (Cook, 1981; Salt, 1992; Thompson and Bordwell, 2003). But what is it that turned cinema into such a success? I propose that in addition to the ripe technological and social context that promoted cross-cultural dissemination, a major drive in the fast and triumphant evolution of cinema is that this form of art uniquely fits, exploits and expands the potential of basic and critical faculties of human brain and cognition. These are *Working Memory* (WM), *Mental Time Travel* (MTT), *Mental Emotional Travel* (MET), and a spectrum of transitions in consciousness manifested in *Dissociative States*. Furthermore, since cinema taps into the above faculties, it can also be exploited as a convenient scientific tool to investigate those faculties and their brain substrates.

## On information systems in the brain

**Figure 1:**
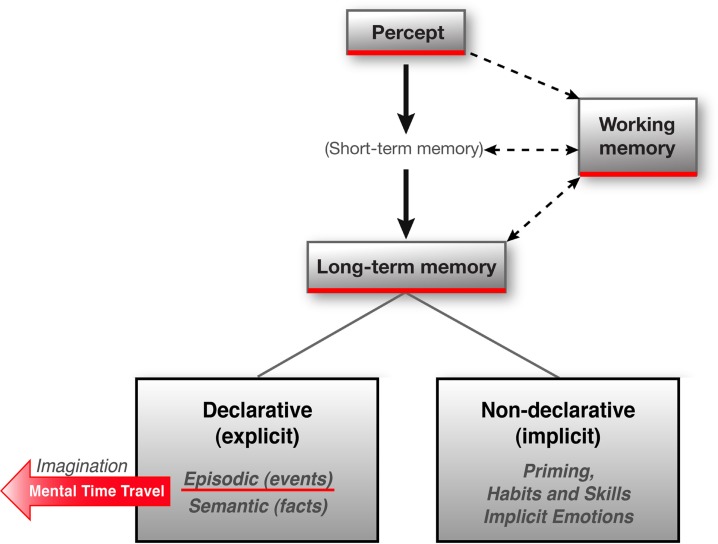
**Memory systems.** Humans have multiple memory systems that could be classified according to multiple criteria. One is time: short- vs. long-term memory. On this axis, working memory (WM) is a type of dynamic short-term memory (see Figure 2A below). Long-term memory (LTM) systems are commonly classified into declarative, i.e., requiring conscious awareness for retrieval, and non-declarative, not requiring conscious awareness for retrieval. Declarative memory is further classified into the memory of events (episodic) and of facts (semantic). Episodic memory is considered to allow mental time travel (MTT) and hence imagining. Non-declarative memory includes types of memory as diverse as priming, habits and skills, motor and emotional reflexes, and more. The declarative—non-declarative dichotomy seems to be honored by the brain, which contains different neural circuits for each system. Only non-declarative (implicit) emotion is noted in the scheme, but mental emotional travel (MET), discussed in the text, involves also declarative (explicit) manifestations. WM, involving attentional control, is usually discussed in the context of declarative tasks, but some information passing via WM is likely to end up over time in non-declarative long-term “stores.” (Adapted from Dudai, 2008).

Understanding how the human brain reads a movie and reacts to it can benefit from understanding how the brain acquires information about the world in general. Our brain has evolved multiple knowledge or memory systems (Figure 1). These are commonly classified along multiple axes (Dudai, 2002). One of these axes is time—whereas some information is stored for seconds or minutes only, other information is stored for weeks, months, years, even a life time. The first type of information is aptly termed “short-term memory” (STM), whereas the second is “long-term memory” (LTM). Another criterion for the taxonomy of memory systems, which is presently dominant in the science of memory literature, concerns the role of conscious awareness in retrieving the information. Hence LTM is considered as either “declarative” (“explicit”) or “non-declarative” (“implicit”) (Dudai, 2002). Declarative memory involves the conscious recollection of facts and events, as opposed to non-declarative, in which retrieval can materialize in the absence of conscious awareness. The declarative—non-declarative dichotomy is widespread in the literature not only because it is intuitively appealing but also because the brain honors it, i.e., different brain circuits subserve the two types of information.

## On working memory (WM)

**Figure 2:**
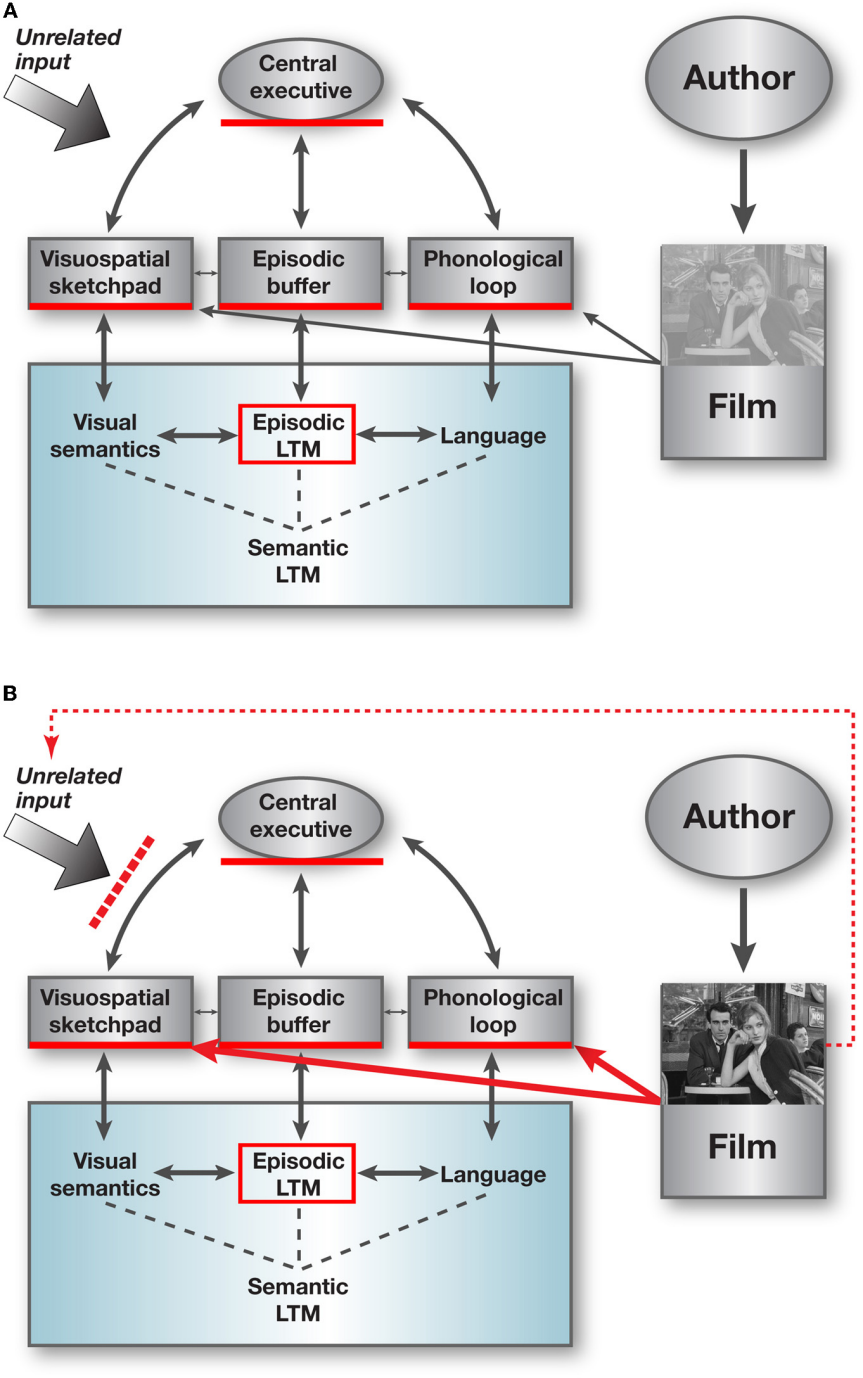
**Film resonates with working memory.** A dominant model of WM considers multiple components (Baddeley, 2007). They are portrayed as a master system, the *central executive*, which executes attentional control over subordinate systems that are content-dedicated mental workspaces, the *phonological loop*, which deals with speech-based information, and the *visuospatial sketchpad*, that deals with visuospatial information. Another postulated component is the *episodic buffer*, in which information from the content-dedicated-workspaces and LTM is temporarily bound under the control of the central executive, to form coherent representations of events, on the potential route to LTM. The mental state evoked by the relevance to survival (e.g., threat, mate, food) of the information flowing into each of the subordinate systems and bound in the episodic buffer, could be considered as “emotion”; it is usually not explicitly included in models of WM and therefore not depicted in the scheme discussed here, yet is highly relevant to the appeal and effect of cinema (see MET in the text). **(A)** Defining attributes of narrative film resonate neatly with multiple components of WM, as well as with effective transformation of information from the episodic buffer into long-term memory. Three major attributes are contextual focusing of the central executive toward the stimulus, intense multi-modal co-activation of both the visuospatial sketchpad and the phonological loop, and compression of narrative highlights that facilitate the focusing of the CE as well the pruning of information to be consolidated from the episodic buffer into LTM. “Author” usually represents multiple individuals though in some cases mainly the director, still never really in isolation. **(B)** Captivating movies can induce a dissociative state in which the movie stimulus dominates the operation of WM components to temporary block simultaneous unrelated input. For further discussion including comparison to other art forms and other dissociative states, see text. (The frame in the inset is from Bresson's *Pickpocket*, 1959) (Adapted from Dudai, 2008).

A dedicated information processing system that combines STM and LTM, is “WM” (Figure 2A). WM is a limited capacity system embedded in distributed brain circuits, that holds information under attentional control in temporary storage during the planning and execution of a task (Miller et al., 1960; Baddeley, 2007). It combines on-line information (i.e., percepts) with off-line information (i.e., LTM) to yield temporary task-oriented internal representations. Some of these representations may subsequently become consolidated into LTM, but often, it is disadvantageous to retain the task-related information in LTM because it may interfere with subsequent tasks. WM is hence a “mental hub” essential for mentation and behavior and indispensible for human cognition and intelligence. Rudimentary WM capabilities may exist in species lower on the phylogenetic scale, but it is considered to have reached its pinnacle in humans, and it takes years to mature in the individual of the species (Luciana and Nelson, 1998).

A particularly influential cognitive model of WM proposes three types of components (Baddeley, 2007) (Figure 2A). One is an attentional control system, termed the “central executive” (CE). Another type is content-dedicated workspaces that are depicted as subordinates of the CE. The model singles out two: a phonological loop, which deals with speech-based information and is assumed to comprise a phonological store and articulatory rehearsal mechanism, and a visuospatial sketchpad, which deals with visuospatial information. The two workspaces are assumed to process information related to the most salient domains of the human mind—vision, space, sound, and language. Additional “workspaces” may exist (Yeshurun et al., 2008). Finally, a third type of hypothetical component is the episodic buffer: mental space in which information from the content-dedicated workspaces and LTM is temporarily bound under the control of the CE to form coherent representations of events, on their potential route to LTM. The CE is postulated to interconnect with modulatory and reinforcing circuits, e.g., encoding emotion and hedonic valence, which control the allocation of attention and filter the transformation of WM representations to LTM.

It is noteworthy that generic attributes of film resonate optimally with the capabilities of WM, and that WM seems to be able to exploit efficiently information in movie stimuli. This resonance was postulated to greatly enhance the rapid successful integration of movies as an “extracorporeal” cognitive organ and a global social phenomenon (Dudai, 2008). Several points support this assumption (Dudai, 2008):

- Cinema is first and foremost a visual art. Humans are visual animals, and therefore are likely to be strongly attracted by visual stimuli. Furthermore, movies depend heavily on motion [movi(ng pictur)e], which is a prime mover of attention. This is expected to strongly and effectively engage the CE and the visuospatial sketchpad.
- The auditory input in movies engages the phonological loop, thus activating the second major mental working space of WM. Coincidence detection of different inputs is considered instrumental in successful encoding at different levels of brain function (Dudai, 2002). The multimodality of film hence enhances its perceptual and mnemonic effectiveness. The unique role of multi-sensory synergism in film has long been noted by major film directors (Eisenstein, 1929). Indeed, some silent films have outstanding affective impact and artistic qualities, nevertheless, activation of the brain's language workspace is likely to occur even in the absence of sound, by observing people talking and trying to decipher what they say. It is also of note that even silent film had snapshots of explicit verbal information, provided by intercalated text slides.
- The multi-modal input of the film stimulus, which engages both the visuospatial sketchpad and the phonological loop, results not only in reinforcement of perceptual and mnemonic encoding by coincidence detection in associative brain circuits, but also permits better exploitation of the inherently limited capacity of WM and hence promotes the processing of a richer percept at any given point in time. This is because the limited capacity of each of the two dedicated workspace systems of WM is independent of each other (Baddeley, 2007).
- The episodic buffer binds ongoing episodes, which then consolidate into LTM (Dudai, 2004). In real-life, modulatory and reinforcing circuits promote consolidation of some but not other pieces of information during the event or immediately afterwards. If the time window of the event is longer than a few hours or days, the saliency and relevance of certain individual event fragments might be disregarded and proactive and retroactive interferences take place (Wixted, 2004). This could result in lack or erroneous binding of the narrative. In the narrative movie, the “author” pre-selects the events to be presented in the movie. As a consequence, the episodic buffer of the spectator ends up receiving a narrative that is already filtered to create the desired effect within the temporal limits of the film. This is expected to facilitate binding and consolidation, hence enhancing perception, reward, and memory.
- Physical time in real life flows unidirectionally. In contrast, compression and inversion of time is an essential device in film art (Tarkovsky, 1986; Turim, 1989). At the level of the brain machinery, this may create a mismatch between what is naturally anticipated and what happens on the screen. Such mismatch is known to augment saliency and attention and enhance encoding (Rescorla and Wagner, 1972). In terms of brain mechanisms, this is expected to be mediated via activation of modulatory circuits linked to WM, and particularly the CE, the episodic buffer, and the consolidation of memory from the latter to LTM. It is tempting to consider the process as a brain correlate of defamiliarization, proposed as an essential artistic device at large (Shklovsky, 1917).
- Similarly, spatial coordinates are also manipulated by the author (using close-up, panning, light effects and cuts), altering attention and defamiliarization (Eisenstein, 1947; Andrew, 1976; Bordwell and Thompson, 2004).
- Attention combined with defamiliarization-induced saliency is also enhanced by the context of the movie spectator, commonly a dark enclosure with other people present but in the absence of explicit social interaction. This could markedly affect the CE, focusing attention and creating a special mind set, and could also activate social-intimacy and safety reward circuits. This enhanced attention in the semi-detached milieu could further activate the episodic buffer, while at the same time promote a transient, mild dissociative state (and see below). This added-value of contextual defamiliarization may account for the failure of Edison's Kinetoscope, in which spectators watched movies in isolation.
- Dissociative states of the aforementioned type can be assumed to involve transient loss of inhibitory control by frontal brain areas, i.e., disruption of CE function. This loss of control is potentially rewarding (as illustrated by the individuals and communities who enter trances of various sorts voluntarily; Kihlstrom, 1985; Robinson and Berridge, 2003). Once induced by spatiotemporal MTT in the unique contextual setting and mental set, the dissociative state in the spectator might be rewarding *per se*, promoting positive feedback that further promotes the enjoyable mental state.

## On mental time travel

Resonance with the capabilities of WM is, however, only one component in the productive dialogue between brain and cinema. Another is the ability of movies to extend, manipulate and promote individual experimentation with another pinnacle of human brain and cognition, namely, MTT (also termed chronesthesia). MTT refers to the ability to be aware of one's past and reenact it in mind, as well as to imagine potential future scenarios (Tulving, 1983, 2005; Suddendorf and Busby, 2005; Bar, 2011; Suddendorf et al., 2011). Some consider this mental faculty to be uniquely human, others posit that rudimentary forms exist in some other species as well (Tulving, 1983, 2005; Suddendorf and Busby, 2005; Bar, 2011; Suddendorf et al., 2011). MTT is the decisive fingerprint of bona-fide episodic memory. Its imagining component, i.e., the ability to mentally construct potential scenarios of future occurrences, has been suggested as a major drive in the evolution of episodic memory (Dudai and Carruthers, 2005; Schacter and Addis, 2011). It may also underlie the feeble veracity of episodic recollection: strict faithfulness to details might hamper useful imagination. It is noteworthy that episodic recollection and imagining share brain circuits (Hassabis and Maguire, 2011; Schacter and Addis, 2011).

Movies promote, entrain and enhance MTT. Their ability to simulate real-life, day-dreaming, and “dream-like” experiences by fusing multimodal perception with emotional and cognitive overtones, distanced from the acute spatiotemporal coordinates in which the spectator is present at that specific point in time, was long noted by movie theorists (Eisenstein, 1969; Morin, 2005). Indeed movies have been recently introduced as effective stimuli in perceptual studies and memoranda in memory studies that combine behavioral analysis and functional neuroimaging (Hasson et al., 2006, 2008a; Furman et al., 2007; Mendelsohn et al., 2008, 2009, 2010). What is less noted in the studies of brain and cognition is that the experience of becoming immersed in a movie also provides an intriguing mental experimentation space for exercising MTT in the observer, and as such, can provide internal reward in exposing the immersed observer to imaginary experiences otherwise unattainable. This rewarding value is shared with other forms of art, however, cinema, being a multi-model art form, may provide a more universal, and for most individuals probably more accessible opportunity, to tap into this type of reward.

## On mental emotional travel

Similarly to the promotion and entraining of MTT, and coupled to this ability, movie art can also be considered an effective manipulator of MET. “Emotion” is considered in the scientific literature in multiple connotations, the two dominant ones being emotions as a trigger of an automatic physiological response, mostly to danger and social cues, and emotions as the subjective feeling which accompanies the above and other states related to the relevance of ambience to the self (LeDoux, 1996). In the present context, it is the latter manifestation of emotion that counts. Given the proper movie, the observer can wander into and explore a spectrum of rich and deep emotional experiences and domains unexplored by most people in daily life, let alone within the condensed time capsule that the movie offers. Selected (admittedly idiosyncratic) examples range from neorealistic cornerstones (e.g., Ozu's *An Inn in Tokyo*, its artistic sequel, *Bicycle Thieves* by De Sica, or Rosselini' *The War Trilogy*) to the bleak and provocatively disturbing postmodernism of Haneke in *Caché* and other masterpieces. Exploration of the unlimited imaginary emotional spectrum further expands the mental reward space provided by cinema. Although the movies and the examples of the cinematic devices brought up in this article mostly refer to “auteur” (in European cinema a top director is considered the author of the movie) or arthouse movies, clearly, a movie need not be a high quality art piece to achieve the aforementioned effects. Any emotional drama, irrespective of its artistic quality and literary value, evokes MET. Indeed, both MTT and MET are generic attributes of the cinema.

## On cinematic approaches to promoting MTT and MET

A wide range of styles used by various film directors can effectively trigger and promote explorative MTT and MET. It is noteworthy that excessive audiovisual effects or mimicking real-life excessively to the point that defamiliarization, an important artistic device (e.g., Brecht, 1977), is minimized, are not necessarily helpful; making a movie too real was proposed to even hamper imagining and hence MTT (Dudai, 2008). In the present context, only a single particularly interesting style, which echoes a highly successful conceptual framework of modern scientific research and therefore might particularly be appreciated by scientists, will be briefly noted. This is reductionism, characterized by an attempt to identify cognitive, emotional, and motor universals and manipulate them in a minimalistic manner. This is a bottom-up approach guided by the goal of entwining emergent cognitive and emotional outcomes from their most basic building blocks. This seems to effectively prompt the observer to reconstruct situations, plots and emotions while maximizing mental effort, attention and self-involvement—not unlike those sometimes required for successful reenactment of remote self-episodes. Two major representatives of this approach come immediately to mind, each unique in his idiosyncratic implementation of the concept: the French auteur Robert Bresson (1901–1999) and the Japanese auteur Yasujirô Ozu (1903–1963).

Bresson (*A Man Escaped, Pickpocket*, *Au Hasard Balthazar, Mouchette*, and nine other masterpieces), a master of lean and crystallized cinematography, recurrently used what he called “models”: non-professional actors trained in neutral line reading, automatic gestures, and emotional inexpressiveness (Quandt, 1998). He attempted to identify and use the most reducible behavioral elements, and strip these motor, cognitive, and emotional atoms from all superfluous context- and time-dependent heuristics. By doing so he wished to present the “pure” human action (and hence potential feelings underlying it) to the observer, and to decipher and reconstruct the scene and its underpinning bare human actions. Bresson's style is to focus on body parts (e.g., hands) rather than the whole body, pushing reductionism even further. “Models who have become automatic (everything weighed, measured, timed, repeated 10, 20 times) and are then dropped in the medium of the events of your film—their relations with the objects and persons around them will be right, because they will not be thought” (Bresson, 1975) “… It is with something clean and precise that you will force the attention of inattentive eyes and ears.”

Ozu, in contrast, exercised reductionism and minimalism while relying on a small cast of professional actors, many of them playing recurrently in his films. The overall outcome in terms of inciting universal responses in the observer is, however, quite similar to that of Bresson, though reflecting a more humane and empathic and less austere and religious ambient than the latter. Ozu used an almost unbelievable number of takes for every scene, “correcting our every inflection, over and over … trying to reduce things to their most basic essence, free of all excess” (Arima, 2003). An idiosyncratic Ozu shooting style, which promotes attention and focuses the gaze, was the so called “tatami shot,” in which the camera (always static, no tracking shots) is placed at a low height, supposedly at the eye level of a person kneeling on a tatami mat. Ozu produced 53 movies, most of them a variant of a similar type of simple plot focusing on family life and generation gaps; *An Inn in Tokyo, The Only Sun, Late Spring* and *Tokyo Story* are notable examples. In a way, Ozu (like many great artists) repeated his leitmotif, again and again, 50 times, each time trying to extract novel nuances using the same elementary building blocks (Bordwell, 1988). Bresson and Ozu, each in his unique reductive and minimalist manner, exposed the underlying unity of the human condition. Their rationale, a driving force for many artistic giants, was effectively expressed two centuries earlier: “Nothing can please many, and please long, but just representations of general nature” (Johnson, 1765).

## On dissociative states

All forms of art are capable of inducing some form or another of transient “dissociative states.” These are disruptions in integrative functions of consciousness, memory, identity, or perception, and can be pathological. Transient, mild dissociation occurs however in normal individuals when they get immersed in some activity while suppressing attention to other external or internal stimuli (see also in this context “suspension of disbelief” in Bazin, 1967). When induced by art, dissociative states could be regarded as the enslavement of the CE of the consumer to that of the author (Dudai, 2008) (Figure 2B). The appreciation that the artist can come to control the audience's mind has of course been with us since classical times, probably dating back to cave art at the dawn of civilization (Lewis-Williams, 2002). Although while engaged in the creative act the artist may not necessarily be aware of the long-reaching effects on the other's mind, many are; a notable example in film is Eisenstein, who, faithful to the tradition of Soviet pragmatism and Pavlovian physiological psychology, attempts to condition the spectator with discrete sensory and semantic devices (Eisenstein, 1947). Tarkovsky formulates the mind-control objective boldly: “… a kind of revision takes place within the subjective awareness … this process is inherent in the relationship between writer and reader; it's like a Trojan horse, in whose belly the writer makes his way into his reader's soul” (Tarkovsky, 1986). Many film theorists noted the dissociative, or “lowered consciousness” state that can be induced by cinema (Kracauer, 1960), some attributing it to the aforementioned “dream-like” state (Clair, 1953) or to “day dreaming” experiences (Morin, 2005). The depth, persistence and quality of the dissociative state clearly depends on the reader, listener or spectator, on the specific work of art, and on the context, but to get an idea, the reader of this discussion might wish to imagine getting absorbed in a book, a quartet, or a film. This transient partial detachment from the outside world is a function of the state of the WM system at that specific point in time. Dissociative states can have a marked reward valence—as well exemplified by those taking drugs to obtain them, risking addiction (Robinson and Berridge, 2003). They hence provide another potential reward value that promotes the enjoyment of movies.

## On cinema as a particular mental experimentation space

One could argue that the ability of cinema to resonate with WM and to promote, instigate and extend MTT, MET, and limited dissociative states, is shared by other forms of art as well. The role in promoting and enriching MTT and MET is encapsulated already in Aristotle's reference to the poet, whose function is: “… to describe, not the thing that has happened, but a kind of thing that might happen … ” (*Poetics* 1451.1). And one could neatly replace “Shakespeare” with “film auteur” in Johnson's praise of the Bard, who “… approximates the remote, and familiarizes the wonderful; the event which he represents will not happen, but if it were possible, its effects would probably be such as he has assigned; and it may be said, that he has not only shewn human nature as it acts in real exigencies, but as it would be found in trials, to which it cannot be exposed … he who has mazed his imagination … may here be cured … by scenes which a hermit may estimate the transactions of the world, and a confessor predict in the progress of the passions” (Johnson, 1765).

However, in my view, none of the other forms of art combines all the attributes of film, although a good piece of art is capable of evoking MET, and probably to a lesser degree MTT, particularly in the trained mind, eye or ear, irrespective of the medium. Painting and sculpture are visual, but do not involve concrete visual motion, auditory stimuli, and dynamic physical time compression. Even ingenious narrative-telling painters such as Poussin only create limited spatiotemporal compression in the mind of the spectator, restricting the appreciation of the limited mental travel which is anchored in a physically static snapshot only to those who invest proper mental effort. Another example is time-travel elicited by literary fiction, in which MTT, if elicited, is often more fragmentary and has to be accumulated over the time span of reading the piece. Music *per se* is not visual (though may evoke visual imagery). Theatre (including opera and some forms of dance) is audiovisual and uses limited spatiotemporal compression, with more restricted potential MTT and fewer technological capabilities than film (e.g., absence of rapidly merged flashbacks, close ups, and panning, unless film is integrated into theatre, opera, dance, or other forms of the visual arts). Furthermore, having human beings in real time on stage may *a priori* limit defamiliarization. Hence although it is an error to try and rank art forms, as the types of emotional and cognitive enrichment and reward that they incite and provide differ by the art form, the art piece, and the participant or consumer, one could still generalize that film as a medium is that art form that integrates the most varied and advanced technologies for mimesis, while at the same time presenting on average the anonymous spectator with most opportunities and, most importantly, lowest threshold to extract idiosyncratic enjoyment. Still, of course, without investing much mental work on top of the movi[ing pictur]e, and without some experience, one cannot fully appreciate a Bresson, Ozu or a Tarkovsky, or the works of many others.

## On cinema as a scientific experimentation space

**Figure 3:**
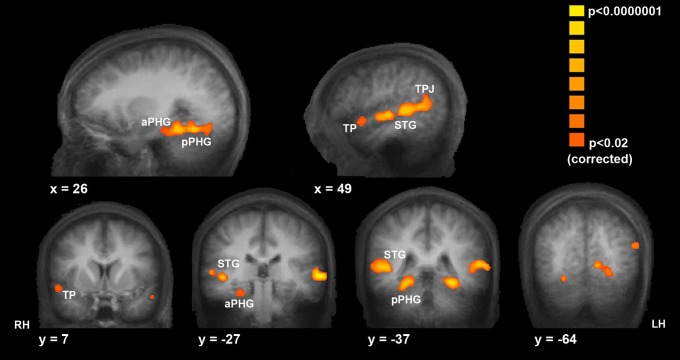
**Film as a window for exploring brain and cognition.** As discussed in the text, movies enrich human cognitive experience, but also provide a window into how this experience is encoded in the experiencing brain, because they can be used as reproducible real-life-like stimuli in perceptual and memory experiments. In this example, Hasson et al. (2008a) used a narrative movie as the stimulus to be encoded in long-term episodic memory. The statistical maps of blood oxygen level dependent (BOLD) activity depict brain areas with significantly enhanced activity during movie events that were subsequently remembered compared to events that were not remembered. These areas include the right temporal pole (TP), bilateral anterior and posterior superior temporal gyrus (STG), bilateral anterior parahippocampal cortex (aPHG), bilateral posterior parahippocampal gyrus (pPHG), and bilateral temporoparietal junction (TPJ). These areas were implicated by other studies in social cognition. RH, LH, are right and left hemisphere, respectively. This suggests that in real-life, the modulation of social cognitive processes impacts episodic memory formation, a finding not commonly unveiled by using simple static and contextless stimuli in memory experiments. (Adopted with permission from Hasson et al., 2008a).

While film equips us with an extracorporeal cognitive and emotional space that can enrich and expand MTT and MET, as well as potentially induce rewarding dissociative states, it also provides scientific research with a powerful tool to probe human brain and cognition. Movies can serve as stimuli and memoranda that can effectively mimic realistic situations (Hasson et al., 2006, 2008a,b; Furman et al., 2007; Mendelsohn et al., 2008, 2010). They permit reproducible presentation of ongoing episodes, and are particularly useful in experiments that involve functional brain imaging, such as fMRI (functional magnetic resonance imaging) (Hasson et al., 2006, 2008a; Mendelsohn et al., 2008, 2010). Indeed the use of movies has already provided novel information on brain processes elicited by complex audiovisual stimuli (Hasson et al., 2006) and on the engagement of identifiable brain circuits in long-term episodic (Hasson et al., 2008a; Mendelsohn et al., 2008, 2010) and autobiographical (Mendelsohn et al., 2009) memory (see also the discussion of “neurocinematics” in Hasson et al., 2008b). Of particular interest is the finding that unlike “traditional” experiments, that consistently unveil subsequent memory effects for still images or context-less verbal material in the mediotemporal lobe (MTL) and the inferior frontal gyrus (IFG), the use of narrative film as memoranda also implicate the superior temporal gyrus (STG), temporoparietal junction (TPJ), and the temporal poles in memory formation (Hasson et al., 2008a) (Figure 3). These regions have been consistently implicated in social cognition and perception; this suggests that in real-life, the modulation of social cognitive processes impacts episodic memory formation, a finding that tended to escape under the radar of brain imaging paradigms using non-realistic memoranda.

All in all, hence, movies can enrich human mental experience, yet can also provide a window into how this experience is encoded in the experiencing brain.

### Conflict of interest statement

The authors declare that the research was conducted in the absence of any commercial or financial relationships that could be construed as a potential conflict of interest.
